# Psychological distress over time among adolescent Soluna users: A prospective observational study

**DOI:** 10.1016/j.invent.2026.101001

**Published:** 2026-09-24

**Authors:** Katherine Cohen, Andy Rapoport, Shannon Hill, Regina Misch, Emily P. Cowling, Louisa Salhi, Jessica L. Schleider

**Affiliations:** a Department of Medical Social Sciences, Feinberg School of Medicine, Northwestern University, 625 N Michigan Ave 21^st^ floor, Chicago, IL, 60608, United States of America; b Kooth Digital Health, 167 North Green Street, Chicago, IL, 60607, United States of America; c Threshold LLC, York, SC, United States of America; d Kooth Digital Health, 5 Merchant Square, London, W2 1AY, GB, United Kingdom; e University of Manchester, United Kingdom

**Keywords:** Digital mental health, Youth, Coaching, Peer community

## Abstract

**Background:**

It is critical to investigate innovative methods of providing immediate mental health support for youth. Soluna is a digital mental health and wellbeing platform for youth ages 13–25 launched and developed through a partnership between the California Department of Health Care Services and Kooth Digital Health as part of California's Children and Youth Behavioral Health Initiative. Soluna provides users with rapid access to self-help content and tools, pre-moderated peer community, coaching sessions, and care navigation from any internet-connected device.

**Objective:**

The purpose of this study is to examine mental health and wellbeing outcomes over time among a self-selected subset of Soluna users aged 14–17.

**Methods:**

We conducted a non-randomized, prospective, observational study. Participants completed three surveys (baseline, one-month, and three-months post-baseline) that assessed psychosocial outcomes and self-reported engagement with platform features. The primary outcome was psychological distress as measured by the YP-CORE. We conducted pre-registered analyses to determine whether outcomes changed among participants over the three-month evaluation period.

**Results:**

A total of 232 participants completed baseline measures, with 136 and 98 participants retained at one- and three-month follow-ups, respectively. Participants showed statistically significant within-group reductions in the primary outcome of distress from baseline to three-months (*d* = 0.63), and statistically significant improvements in secondary outcomes of depression, anxiety, hopelessness, loneliness, suicidal ideation, and quality of life (*r* = 0.35–0.53).

**Conclusions:**

In this prospective observational study, participants showed statistically-significant within-person reductions in distress. Findings point towards the potential that scalable digital platforms like Soluna may offer accessible mental health and wellbeing support for youth.

## Highlights

• Soluna is a digital mental health and wellbeing platform available for free for youth in California
• Across a 3-month period, Soluna users saw reductions in psychological distress
• Changes in distress were not predicted by participants' sociodemographic and historical factors

## 1 Introduction

Youth in the United States (US) are facing extraordinary degrees of mental distress. National data from the Centers for Disease Control and Prevention suggest that in 2023, 40 % of youth in the US experienced persistent feelings of sadness or hopelessness and 20 % seriously considered attempting suicide (Centers for Disease Control and Prevention, 2024). Unaddressed mental health concerns in youth can lead to long-term impairments in functioning (Dooley et al., 2023; Thompson et al., 2023); for example, greater mental health concerns are associated with worse academic outcomes (Pagerols et al., 2022), and adolescents categorized as “depressed” are more likely to face unemployment in adulthood (Germinario et al., 2022). The majority of mental health disorders onset before the age of 24, further underscoring the imperative to improve mental health during adolescence (Kessler et al., 2005). Accordingly, experts have called for a population-based, public health approach to address the youth mental health crisis (Dodge et al., 2024; Uhlhaas et al., 2023). Providing psychoeducation and basic socio-emotional skills training to youth could prevent the onset or deterioration of mental health disorders (Sandler et al., 2014; Werner-Seidler et al., 2021).

One pathway for scalable support includes leveraging technology to provide widespread access to evidence-based resources. Youth report interest in digital support due to its flexibility and availability on-demand (Gibson et al., 2016). Digital programs may be particularly beneficial for youth in marginalized communities whose needs are not met by traditional services (Schueller et al., 2019). For example, LGBTQ+ youth may seek support through digital tools because of challenges finding a culturally competent and accepting clinician (Lucassen et al., 2015). Digital programs are not only acceptable among youth but may also be effective. For example, meta-analyses show that youth who participate in internet-delivered Cognitive Behavioral Therapy (iCBT) programs experience reductions in anxiety that are comparable to those in in-person programs (Aspvall et al., 2021; Rabner et al., 2025). A meta-analysis including 1706 adolescents and young adults participating in randomized controlled trials of mental health apps found that app users had significant reductions in depression symptoms compared to participants in wait-list or attention control conditions (i.e., conditions with minimal clinical content that seek to control for nonspecific effects of an intervention; Leech et al., 2021). In addition to reducing mental health problems, literature suggests that self-guided digital programs can be used to promote wellbeing. A systematic review including 1460 youth found that self-guided digital interventions (including apps, web-based programs, computer-based games, and online blogging programs) had positive effects on outcomes such as self-esteem, emotional self-awareness, and problem-solving skills (Babbage et al., 2022).

Both guided and unguided (i.e., self-guided) digital programs have been developed and tested. In guided programs, a trained individual, often called a “coach,” has synchronous or asynchronous contact with users (e.g., chat messages, phone calls) to teach them skills, guide them through program content, provide supportive accountability, or answer questions (Mohr et al., 2011). Literature suggests that guided programs confer more favorable clinical outcomes and lead to greater program adherence compared to unguided programs (González-Robles et al., 2024). However, the scalability of guided programs can be challenging if it is not feasible to provide ongoing human support for every user. A combined approach wherein users can complete self-guided activities on their own but have on-demand access to human support if needed, could provide a balance between efficacy and feasibility. To implement such an approach, it is important to consider how users may benefit from brief, stand-alone interactions with a coach.

There is increasing research on the utility and efficacy of One-At-A-Time (OOAT) approaches and Single Session Therapies (SSTs), which involve an interaction with a trained facilitator that is intentionally designed to elicit change within one session (Dryden, 2020). Although they are facilitated such that clinical change may occur if only one session is delivered, clients are not limited to one session (i.e., one-at a time, rather than one-and-done). They rely on a strength-based, client-led approach to help a client identify “in-session” goals. Results from a systematic review of eighteen studies show that SSTs can effectively reduce mental health symptoms (Bertuzzi et al., 2021). Expanding access to SSTs may prove beneficial, particularly in the context of digital programs, as they allow for human support without requiring long-term engagement. Further, to enhance scalability, trained lay-providers may be leveraged rather than relying on highly-trained clinicians to guide users. Research shows that in the context of providing support in digital mental health programs, lay-providers are equally as effective as licensed clinicians (Titov et al., 2010).

Other methods of receiving human support, such as peer support, could be effective and scalable. Providing and receiving peer support is an evidence-based method for improving mental health, particularly during adolescence when peer influence is highly salient (Fortuna et al., 2022; Tomé et al., 2012). Peer support can take place formally (e.g., trained peer specialists) or informally (e.g., posting and responding to threads in an online community). Research shows comparable effect sizes for formal and informal peer support on mental health outcomes (Yeo et al., 2025). In asynchronous online peer support communities, youth in any location can connect with others who understand their experiences, offer insights for managing symptoms, and learn strategies that work for their peers. Asynchronous peer support communities are popular among youth; in 2019, nearly one-third of youth reported using online mental health communities to manage their mental health, with estimates likely increasing since the pandemic (Cohen et al., 2021).

Digital peer support shows promising effects in research studies, but real-world implementation is more complex. A meta-analysis including 52,703 participants across 73 studies found that digital peer support interventions significantly improved mental health outcomes (Yeo et al., 2025). Unfortunately, online peer support and communication platforms outside of research settings often are not moderated by trained professionals, and some users report that they have been exposed to explicit or aggressive content on online peer support communities (Ruiz, 2024). Some platforms have reported success in maintaining boundaries and safety through careful moderation. For example, the Kooth online mental wellbeing website in the United Kingdom has a peer community support feature that is moderated by trained Emotional Wellbeing Practitioners. In a pilot evaluation, participants who engaged in the peer community support features saw statistically significant reductions in psychological distress and reported in interviews that it was beneficial to relate to other youth with mental health concerns, hear their advice, and provide reciprocal support (Stevens et al., 2022).

Digital programs with human support, single-session approaches, and moderated peer support communities each have evidence demonstrating their efficacy in reducing mental health concerns, but further research is needed to understand their efficacy during real-world implementation. It is critical that these supports are evidence-based, have appropriate safeguards, and are tailored to youth. To leverage the promise of digital tools, an increasing number of digital mental health companies have introduced apps that integrate these approaches to support youth mental health. However, the vast majority of digital mental health companies do not test the effectiveness of their products through research, resulting in the dissemination of mental health tools that are often not effective (Fitzpatrick et al., 2024). It is critical to conduct studies to determine if dissemination of, and investment in, these products should continue. Academic-industry partnerships, wherein researchers at an academic institution collect and analyze data to test companies' products, may be one path towards ensuring that products are evidence-based. At the same time, a common problem within academic research on digital mental health programs is that studies are often conducted in highly controlled research environments rather than in real-world environments (Lyon et al., 2020). To ensure external validity, it is crucial to study digital mental health programs within typical delivery conditions.

## 2 Kooth Digital Health and Soluna

Launched on January 1, 2024, in California, Soluna is a free, confidential digital mental health and well-being platform for youth aged 13 to 25, regardless of insurance coverage or immigration status (Soluna, 2025). Soluna was developed through a partnership between Kooth Digital Health and California Department of Health Care Services (DHCS) as a part of the California's Children and Youth Behavioral Health Initiative CYBHI, a key component of California Governor Newsom's Master Plan for Kids' Mental Health (2022). Soluna was co-designed with input from over 500 California-based organizations, youth, behavioral health experts, and continues to be enhanced through ongoing feedback from a Youth Advisory Board and Scientific Advisory Board (California Health and Human Services Agency, 2026). Soluna is grounded in evidence-based theoretical frameworks (Cowling et al., 2025), incorporating elements of cognitive behavioral models, such as self-determination theory (Ryan and Deci, 2000) and acceptance and commitment therapy. All features in Soluna are anchored to one or more constructs in the platform's theory of change framework, which articulates how skill-building, psychological flexibility, and enhanced socio-emotional functioning are expected to influence both individual and population-level outcomes (Cowling et al., 2025). Soluna is a universal wellbeing service; it is available to all youth aged 13–25 in California regardless of whether they are currently experiencing mental health symptoms. Soluna also includes built-in onramps to targeted support based on individual needs or risk identification. Soluna offers a range of supports for skill development, including educational content and emotion-regulation tools, a safe pre-moderated peer community, behavioral health coaching, and care navigation. Specific features of Soluna are described in detail in the methods section below. The platform aims to foster psychological flexibility by strengthening users' sense of autonomy, relatedness, mastery, self-efficacy and hope. Within the theory of change, building psychological flexibility serves as a key mediator of impact on individual outcomes and population health (Cowling et al., 2025).

### 2.1 The current study

The current study was formulated through a partnership between Kooth Digital Health and the Lab for Scalable Mental Health at Northwestern University. The purpose of this study is to report observational changes in psychosocial outcomes over three months among adolescent Soluna users in California. The pragmatic design allowed for the collection of data during real-world implementation. This study provides an example of a cross-sector partnership that brings together the resources and expertise of both academic and industry experts. The data collection and analysis were completed independently and externally by the Northwestern team. Our primary outcome of interest is overall distress as measured by the Young Person's CORE (YP-CORE; Twigg et al., 2009). We chose this measure because it is transdiagnostic (i.e., youth may experience psychological distress regardless of whether or not they have a mental health diagnosis) and focuses on daily functioning (e.g., sleep, coping, ability to talk to others). This allowed us to examine changes in psychosocial outcomes at a population level in line with Soluna's universal approach. The YP-CORE has also been shown to be sensitive to change in a clinical youth sample (Twigg et al., 2016). We preregistered the following hypotheses:

*Hypothesis 1a (primary outcome):* We hypothesized that youth would show significant (adjusted *p* < .05) decreases in overall distress (YP-CORE scores) from baseline to 1 month, from 1 month to 3 months, and from baseline to 3 months.

*Hypothesis 1b (secondary outcomes):* We hypothesized that youth would show significant (adjusted *p* < .05) decreases in depression symptoms, anxiety symptoms, hopelessness, mental illness stigma, suicidal ideation, and loneliness; significant increases in perceived social support, outcome expectancies for mental health support, and quality of life from baseline to 1 month, from 1 month to 3 months, and from baseline to 3 months.

*Hypothesis 2 (demographic and historical predictors):* We hypothesized that youth would show similar changes in distress, regardless of sociodemographic or historical factors (grade, gender identity, sex assigned at birth, sexual orientation, race/ethnicity, history of adverse childhood experiences, history of bullying).

## 3 Methods

This study is a non-randomized, pragmatic, prospective observational study. The protocol and all study materials were approved by the Institutional Review Board at Northwestern University (#STU00220789) and by the Committee for the Protection of Human Subjects (#2025–011). The study was also approved by the California Department of Health Care Services' Data and Research Committee. Study procedures were pre-registered prior to data collection (https://osf.io/8v4mq). All study activities were conducted virtually through Qualtrics. Data collected through Qualtrics was accessible only by the Northwestern University research team. Participants completed a total of three surveys (baseline, one-month post-baseline, and three-months post-baseline). Participants in this study were not given instructions regarding how often or in what manner they should use Soluna.

### 3.1 Recruitment

Participants were recruited via Soluna between April 28, 2025, and June 27, 2025. A short advert and link to the research study was featured within the platform's content library, displayed in a featured carousel alongside other curated content pieces. Users also received a message about the research study via the platform's notification inbox (but not as a push alert). Individuals who clicked on the study link were automatically re-directed to a web page on Qualtrics that contained information about the study and a link to the screener to determine eligibility. Choosing not to respond to the advert or participate in the study had no effect on access to Soluna. We employed an upper limit of 500 participants total and an upper limit of 50 participants per week to ensure there was sufficient Anonymous Institution staff capacity to implement safeguarding procedures when risk was identified. Participants received the one-month and three-month survey invitations via email, with two reminder e-mails for each follow-up. Participants received a $10 USD gift card after completion of each of the three surveys (up to $30 USD total). The baseline survey was completed between April–June 2025, the one-month survey was completed between May–August 2025, and the three-month survey was completed between July–October 2025.

### 3.2 Sample

Eligibility criteria for this study included being between the ages of 14 and 17, reporting proficiency in reading English, reporting access to the internet (through computer, phone, smartphone, etc.), being registered as a user in California where Soluna is available for youth, sharing an email address (for compensation) as well as a phone number and a parent/guardian phone number (which were to be used if responses indicated risk, as detailed below), and passing the capacity to consent assessment (as detailed below). We chose to limit our sample to youth aged 14–17 to focus on the adolescent developmental period, as compared to the overall Soluna population of those aged 13–25, which spans a larger range of development. Additionally, we excluded any participants from analysis who failed to pass integrity measures, specifically by not answering “Option 3” to a question that asked, “Please select option 3” or by completing the survey in less than five minutes.

### 3.3 Consent procedures

All participants completed an informed consent form that was written at the sixth grade reading level. Additionally, all participants were required to pass a capacity to consent assessment. The capacity to consent assessment included three multiple-choice questions about what would be involved in the study. For example, one question was, “What happens if I no longer want to participate in the study?” and the correct answer was “It will be no issue if you want to leave the research; we'll keep your information, but we won't if you tell us not to.” Participants were given two chances to answer all three questions correctly. Participants who were eligible based on screening questions and who passed the capacity to consent questions were asked whether they would like to participate. If they selected, “I agree,” they were asked to provide an electronic signature and then continued to the baseline questionnaires. If they selected, “I am not sure” they were encouraged to contact the research team with questions and advised not to participate if they were hesitant. For one week, these participants were permitted to return to the consent form where they left off. Participants who were ineligible were informed that they were not eligible and directed to a list of mental health resources. Youth ages 13 to 25 in California are able to access and use Soluna without requiring parental consent. We received a waiver for parental consent for this study to align with this policy, within each approval process - Northwestern University IRB and from CPHS.

### 3.4 Soluna

Soluna is available for free to all California youth and young adults aged 13 to 25, without the need for referral, insurance coverage, diagnosis, disclosure of immigration status, or parental consent (consistent with California State law, which permits minors aged 12 and older to consent to outpatient mental health treatment or counseling services when certain conditions are met; Justia Law, 2025). Soluna is promoted through existing outreach channels, including partnerships with schools, health care providers, and community-based organizations; participation in local community events; and leveraging trusted peer and community networks to increase awareness and engagement. In parallel, digital promotion is delivered through social and paid media campaigns, complemented by print materials, such as posters, flyers and billboards distributed in school, college, and community settings.

Soluna includes self-guided content and well-being tools, a pre-moderated peer community, a repository of searchable resources, one-to-one coaching sessions with certified behavioral health professionals, and care navigation to higher acuity care and additional community resources. Soluna is available in English and Spanish. Coaching, care navigation, and user support is available in 17 Medi-Cal Threshold languages through translation services.

Self-guided content and well-being tools are described in more detail in a previous publication (Yourell et al., 2026). Soluna's anonymous, pre-moderated peer community facilitates safe peer-to-peer engagement through polls, prompts, and forums. All content undergoes review by a trained specialist before being published and made visible to other users. This pre-moderation process ensures that posts align with community guidelines, legal and regulatory compliance, and best practice guidelines to ensure safety and relevance. Service users cannot directly message each other; all peer interactions occur within the moderated forums.

Professional human support within Soluna is delivered through two service types: behavioral health coaching and care navigation. Behavioral health coaching is provided by three categories of trained professionals: (1) certified peer support specialists, who draw on lived experience of behavioral health recovery to provide peer-based support; (2) mental health support specialists, who deliver brief, skills-based coaching focused on emotional wellbeing, coping strategies, and personal growth, and (3) substance use disorder (SUD) counselors, who provide coaching and support related to substance use concerns. Although these coaching roles share functions such as supportive listening, goal setting, psychoeducation, and skills development, each contributes specialized expertise based on training and experience. Care navigation is provided by care coordinators, whose primary role is to help users identify resources, access referrals, and connect with appropriate community, behavioral health, and social support services.

Users may access behavioral health coaching through either scheduled appointments or drop-in sessions during available service hours (10:00 a.m. to 10:00 p.m. Pacific Time) and may choose to engage via text, video, or phone. Depending on user preference, presenting needs, and staff availability, users may either select or be matched with an appropriate support professional. Care navigation services can be accessed when users require assistance connecting with external resources, referrals, or treatment services. This structure allows users to receive support that aligns with their goals and needs while ensuring access to specialized expertise when appropriate.

Clinical Governance and Safeguarding oversight is provided by licensed clinical professionals (LCPs), under the leadership of clinical psychologists. LCPs offer real-time consultation and clinical risk assessment support to practitioners, providing situational guidance, de-escalation strategies, and safety planning when concerns arise. They oversee escalations to statutory agencies and coordinate connections to appropriate local intervention or emergency services in line with established privacy and safeguarding protocols. LCPs uphold clinical quality through audit and review processes in collaboration with clinical leads, adherence to documented practice standards, participation in supervision and training, and proactive identification of risks or improvement opportunities to strengthen safe, consistent service delivery across the platform.

Soluna uses a solution-focused, person-centered “one-at-a-time” model informed by Single Session Therapy (SST). Soluna's model is designed to focus on the concern identified by the user at the outset through the Session Wants and Needs Outcome Measure (SWAN-OM; De Ossorno Garcia et al., 2023), a brief survey given to users at the outset of a coaching session. Soluna embeds a comprehensive clinical safety framework. It combines technology, licensed clinician oversight, and a suite of robust protocols to detect and respond to signs of risk early, with a focus on proactive and continuous improvement.

Soluna offers care navigation as a concierge-style approach to connecting youth with free or low-cost services in their community, or services covered by the users' insurance if such information is available, including healthcare as well as support for other Social Determinants of Health including food and housing support. Care Coordinators use Soluna's Affiliate Network, a curated database of vetted clinical providers and community resources who can receive closed-loop referrals from the Soluna Care Coordinator team. Coordinators can assist with insurance verification, appointment scheduling, and continuity of care.

### 3.5 Measures

#### 3.5.1 Demographics

Demographic information was collected during the baseline survey. Participants were asked to report their sex assigned at birth, gender identity, sexual orientation, race/ethnicity, primary language, zip code, and grade. Participants were permitted to select multiple options for gender identity and race/ethnicity.

Participants additionally provided their email address (for compensation and follow-up surveys), their phone number (in case of risk, additional detail below), and their parent/guardian's phone number (in case of risk, additional detail below). Participants were allowed to select “prefer not to respond” to all demographic questions besides their phone number and their parent/guardian's phone number.

#### 3.5.2 Mental health questionnaires

##### 3.5.2.1 Primary outcome: Young person's CORE (YP-CORE)

This 10-item scale asks participants to indicate how often they experience symptoms of distress over the last week (e.g., “I've felt edgy or nervous”) on a 5-point Likert scale ranging from 0 (Not at all) to 4 (Most or all of the time). Scores are summed and range from 0 to 40, with higher scores indicating greater levels of distress (Twigg et al., 2009). For the purposes of exploratory analysis, scores greater than or equal to 14.1 were considered clinically-elevated (including all age bands and genders; Twigg et al., 2016). The YP-CORE was completed at all three timepoints. Internal consistency was α = 0.79 at baseline, α = 0.82 at one month, and α = 0.82 at three months.

##### 3.5.2.2 Patient health questionnaire-8 (PHQ-8)

This 8-item scale asks participants how often they have experienced symptoms of depression (e.g., “Little interest or pleasure in doing things”) over the last two weeks on a 4-point Likert scale ranging from 0 (Not at all) to 3 (Nearly every day). Scores are summed and range from 0 to 24, with higher scores indicating greater levels of depression (Kroenke et al., 2009). The question concerning suicide that would be in the PHQ-9 was not included because suicidal ideation was assessed through other metrics. The PHQ-8 was completed at all three timepoints. Internal consistency was α = 0.83 at baseline, α = 0.83 at one month, and α = 0.87 at three months.

##### 3.5.2.3 Generalized anxiety questionnaire-7 (GAD-7)

This 7-item scale asks participants how often they have experienced symptoms of anxiety (e.g., “Feeling nervous, anxious or on edge”) over the last two weeks on a 4-point Likert scale ranging from 0 (Not at all) to 3 (Nearly every day). Scores are summed and range from 0 to 21, with higher scores indicating greater levels of anxiety (Spitzer et al., 2006). The GAD-7 was completed at all three timepoints. Internal consistency was α = 0.86 at baseline, α = 0.89 at one month, and α = 0.91 at three months.

##### 3.5.2.4 Beck hopelessness Scale-4 (BHS-4)

This 4-item scale asks participants to indicate their current level of hopelessness through rating their agreement with statements (e.g., “My future seems dark to me”) on a 4-point Likert scale from 0 (absolutely disagree) to 3 (absolutely agree). Scores are summed and range from 0 to 12, with higher scores indicating greater levels of hopelessness (Perczel Forintos et al., 2013). The BHS-4 was completed at all three timepoints. Internal consistency was α = 0.86 at baseline, α = 0.85 at one month, and α = 0.87 at three months.

##### 3.5.2.5 UCLA loneliness scale-3 (ULS-3)

This 3-item scale asks participants to indicate how often statements reflecting loneliness (e.g. “I feel left out”) are descriptive of them on a 4-point Likert scale from 1 (Hardly Ever) to 3 (Often). Scores are summed and range from 3 to 9, with higher scores indicating greater levels of loneliness (Hughes et al., 2004). The ULS-3 was completed at all three timepoints. Internal consistency was α = 0.78 at baseline, α = 0.77 at one month, and α = 0.83 at three months.

##### 3.5.2.6 Patient-reported outcomes measurement information system Global Health short form (PROMIS 10)

The PROMIS-10 Global Health scale is a validated, 10-item instrument designed by the NIH PROMIS initiative to assess general domains of health-related quality of life. The PROMIS-10 Global Health measure was completed at all three timepoints. There are 2 summary scores for the PROMIS: one for mental health and one for physical health. Raw summed scores are converted to T-scores and categorized according to T-score cut points into the following categories: poor, fair, good, very good, and excellent (Hays et al., 2009; HealthMeasures, 2021). The general population mean is 50 with SD of 10.

##### 3.5.2.7 Suicidal ideation attributes scale (SIDAS)

This 5-item scale assesses suicidal ideation. The first question asks participants, “In the past month, how often have you had thoughts about suicide? (0 = Never, 10 = Always).” If participants responded with a score above zero, they were asked the second question, “In the past month, how much control have you had over these thoughts? (0 = No control, 10 = Full control).” If they responded to the first question with zero (never had thoughts about suicide), question 2 was skipped and automatically scored as zero. Questions 3–5 ask additional questions regarding suicidal ideation on a ten-point likert scale. Scoring includes reverse-coding question 2 such that less control over thoughts indicates greater risk. Summed scores then range from 0 to 50 with higher scores indicating greater frequency and negative impact of suicidal ideation (van Spijker et al., 2014). The SIDAS was completed at all three timepoints. Internal consistency was α = 0.88 at baseline, α = 0.94 at one month, and α = 0.91 at three months.

##### 3.5.2.8 Self-harm question

Participants responded either “Yes” or “No” to the question: “In the past month have you ever deliberately hurt yourself or done anything that might have harmed you or even killed you?” (Moran et al., 2012). The Self-Harm Question was completed at all three timepoints.

##### 3.5.2.9 Multidimensional scale of perceived social support (MSPSS)

This measure assesses perceived levels of social support from family, friends, and significant others. There are 12 items in the original questionnaire that can be separated into three subscales, each made up of 4 items, independently assessing perceived support from family, friends, and significant others. In this study, we are only using the “family” and “friends” subscales. Responses to items are on a 7-point Likert scale ranging from 0 (“very strongly disagree”) to 6 (“very strongly agree”). Higher scores indicate higher levels of perceived social support from each source (Zimet et al., 1988). The MSPSS was completed at all three timepoints. Due to an error in survey creation, the MSPSS was presented twice in the baseline survey. Scores from the first set of questions were used. Internal consistency for the “friends” subscale was α = 0.92 at baseline, α = 0.95 at one month, and α = 0.94 at three-months. Internal consistency for the “family” subscale was α = 0.93 at baseline, α = 0.92 at one month, and α = 0.89 at three months.

##### 3.5.2.10 Attitudes about mental illness and its treatment scale (AMIS)

This 11-item assessment measures mental health stigma towards others (Kobau et al., 2010). It asks participants to rate how much they agree with statements such as “I believe a person with mental illness is a danger to others” on a five-point unipolar scale (Strongly disagree - Strongly Agree). There are two subscales: Negative Stereotypes and Recovery and Outcomes. The AMIS was completed at all three timepoints. Internal consistency for the Negative Stereotypes subscale was α = 0.68 at baseline, α = 0.74 at one month, and α = 0.76 at three-months. Internal consistency for the Recovery and Outcomes subscale was α = 0.58 at baseline, α = 0.63 at one month, and α = 0.61 at three months.

##### 3.5.2.11 Expectancies

Participants' expectancies regarding mental health treatment were assessed using one item: “On a scale from 1 (not at all helpful) to 10 (extremely helpful), how helpful do you think talking to a professional would be in reducing mental health problems, if you struggle/were to struggle with them?” (Schleider and Weisz, 2018). The Expectancies question was completed at all three timepoints.

##### 3.5.2.12 Bullying

We included two questions from the Youth Risk Behavior Surveillance System (YRBSS) related to bullying: “Have you ever been bullied on school property? Yes or No” and “Have you ever been electronically bullied? (Count being bullied through texting, Instagram, Facebook, or other social media.) Yes or No” (Mpofu et al., 2023). Questions related to in-person and virtual bullying were combined to create a binary history of bullying variables. A “yes” answer to either in-person or virtual bullying indicated bullying history (1). A “no” answer to both questions indicated no history of bullying (0). The Bullying questions were completed at all three timepoints.

##### 3.5.2.13 Multisession services

Participants were asked, “In the last [one/two] months, did you see a mental health professional more than once? Yes or No.” If participants selected “yes” they were asked, “Did you see this person through Soluna or outside of Soluna?” Questions related to multisession services were only asked at the one-month and three-month post-baseline timepoints.

##### 3.5.2.14 Adverse childhood experiences (ACEs) scale

The ACEs questionnaire asks about exposure to violence, childhood emotional, physical, or sexual abuse, and household dysfunction during childhood. Participants completed the ACEs questionnaire at the baseline assessment only, with the time frame of whether they have had each experience in their lifetime [yes/no]. There are ten items (Felitti et al., 1998).

#### 3.5.3 User engagement & satisfaction

##### 3.5.3.1 Qualitative feedback

At the one-month and three-month post-baseline assessments, participants were asked to respond to the following open-ended questions: (1) What are some things you liked about Soluna? (2) What are some things you would change about Soluna? (3) Anything else you would like to share about your experience of Soluna?

##### 3.5.3.2 Self-reported user engagement

At the one-month and three-month timepoints, participants were asked, “What parts of Soluna have you used?” Participants could select any of the following (and could select more than one): I've read, listened to, or watched content (e.g., a story or scenario), I've used a tool (e.g., free write, mood log, or thought shaker), I've responded or posted in the community space (e.g., a poll, forum, or live event), I've chatted with a coach, I've made use of the emergency or crisis services, I've made use of the directory or additional services (e.g., food or housing), or None yet, I've only had a look around.

### 3.6 Risk procedures

If participants endorsed a non-zero response to any item on the SIDAS scale (besides one question that was reverse coded, in which case a non-ten response was indicative of risk), or a “yes” answer to the self-harm question, or a non-zero answer to item 4 of the YP-CORE (“I've thought of hurting myself”), they were immediately presented with mental health resources, including 988 and other crisis supports, in addition to other well-being resources, with specific lists for People of Color and LGBTQ+ friendly supports. They then received an email explaining that they would be contacted by a member of the research team within 24 h. A member of the research team who was trained in conducting risk assessments reached out to the participant within 24 h. The team member first sent a text message to the phone number to inform the participant that they would receive a phone call in one hour unless they responded to indicate that they believed the risk flag was an error. If the participant did not respond within an hour, the team member called to conduct an assessment to detect imminent risk. If participants did not answer their phone, the team member left a nondescript voicemail (to preserve participant privacy), emailed the participant again, and attempted to call again within the 24 h period. Two voicemails were to be left, followed by an email. These procedures were the same whether a participant completed the survey fully or stopped midway through a survey. Team members checked for incomplete responses at least once per day.

During risk assessments, participants were asked, “On a scale of 1–10 (1 = not at all and 10 = completely), how safe do you think you can keep yourself after this call?” If participants responded with “10” they were given a list of mental health resources. If participants responded below 10, additional questions were asked to determine if the risk for harm was imminent. If imminent risk was detected, the youth's parent or guardian was contacted as well as emergency services. Participants were informed in the consent form, and again when providing their parent/guardian's phone number, that their parent/guardian would only be contacted if they were in immediate danger. If imminent risk was not detected, the youth completed a safety plan with the team member. This procedure is in line with previous studies conducted by the research team.

### 3.7 Data analysis

#### 3.7.1 Pre-registered analyses

To test hypotheses 1a and 1b at the one-month follow-up, we conducted either paired *t*-tests or Wilcoxon signed-rank tests. We first tested the assumption of normality (via the Shapiro-Wilk normality test). If the assumption was met, we conducted a paired *t*-test. If the assumption was not met, we conducted a Wilcoxon signed-rank test. We originally did not pre-register the SIDAS as a secondary outcome but elected to include it in the current analyses. Additionally, we originally pre-registered that we would test the Total Difficulties scores on the Strengths and Difficulties Questionnaire (SDQ) as a secondary outcome (Goodman, 2001); however, results showed that the internal consistencies for the Total Difficulties score and the other subscales of the SDQ were unacceptable. Our team therefore elected to report the analyses using the SDQ in the supplementary materials rather than the manuscript.

To test hypothesis 2 at the one-month follow-up, we conducted multiple linear regressions: the outcome variable was one-month YP-CORE scores, and predictor variables included baseline YP-CORE, grade, gender identity, sex assigned at birth, sexual orientation, race/ethnicity, history of bullying, and number of ACEs. Sex assigned at birth was dummy coded, with “male” as the reference group. Gender identity was dummy coded, with “man/boy” as the reference group. Individuals who selected identities other than exclusively “man/boy” or “woman/girl” were coded as “transgender or gender diverse (TGD).” Sexual orientation was dummy coded, with “heterosexual/straight” as the reference group. Race/ethnicity was dummy coded, with “White/Caucasian” as the reference group. Participants who select multiple racial identities were coded as “multiracial.” We did not include language due to only a small number of participants indicating a language other than English. We pre-registered that we would include age and history of previous psychotherapy as potential predictors in hypothesis 2. However, we did not ultimately collect data on previous psychotherapy due to a clerical mistake. The data we collected on age asked participants to choose whether they were 13 or younger, 14–17 (eligible), and 18 or older, therefore exact age could not be included. We included grade as a potential predictor in lieu of age. Additionally, we included sex assigned at birth and sexual orientation as potential predictors. To test hypothesis 2 at the three-month follow-up, we conducted a repeated-measures ANCOVA (Analysis of Covariance).

To test hypothesis 1a and 1b at the three-month follow-up, we conducted either a one-way repeated measures ANOVA (Analysis of Variance) or a Friedman test. We first tested the assumption of normality (via the Shapiro-Wilk normality test). If the assumption was met, we conducted a one-way repeated measures ANOVA. If the ANOVA was significant, we performed post-hoc pairwise comparisons using paired *t*-tests, adjusted using the Bonferroni multiple testing correction method. If Mauchly's test of sphericity was significant, we reported the Greenhouse-Geisser correction. If the assumption of normality was not met, we conducted a Friedman test. If the Friedman test was significant, we performed post-hoc pairwise comparisons using paired Wilcoxon signed-rank tests, adjusted using the Bonferroni multiple testing correction method.

Because this is a pragmatic study including no randomization, we used listwise deletion in all analyses. Statistical significance was set at *p* < .05 (two-tailed). To account for multiple comparisons, we corrected *p*-values using the Benjamini & Hochberg correction (Benjamini and Hochberg, 1995).

#### 3.7.2 Exploratory analysis

To compare baseline characteristics and clinical severity between retained and lost participants, we dummy-coded completion of follow-up questionnaires at each time point (0 = did not complete, 1 = completed). We then conducted chi-square tests for categorical variables (e.g., race, gender) and either paired *t*-tests or Wilcoxon signed-rank tests for continuous variables (e.g., baseline distress). To account for multiple comparisons, we corrected *p*-values using the Benjamini & Hochberg correction.

We tested whether the primary outcome of distress significantly changed over the three-month period among a subset of users who had clinically elevated distress at baseline. We used the same analytical methods as in hypotheses 1a and 1b. This analysis was not pre-registered. Additionally, we categorized participants based on whether they showed reliable changes in the primary outcome of distress (YP-CORE). According to Twigg et al. (2016), the overall reliable change index value for the YP-CORE is 7.9 (including all age bands and genders). We additionally conducted a thematic analysis on responses to the open-ended questions (Riessman, 1993).

As a sensitivity analysis, we ran 13 independent mixed models for repeated measures (MMRM; one per outcome) which utilized all available outcome observations. Time was modeled as a categorical variable with three levels (baseline, 1 month, 3 months). Within-participant covariance was represented using an unstructured covariance matrix (wherein each time point can have a different variance, and each pair of time points can have a different correlation), and model parameters were estimated using restricted maximum likelihood. Model-estimated marginal means and pairwise contrasts were used to evaluate change between time points. Bonferroni correction was employed within outcome contrasts to correct for multiple hypotheses. Importantly, this approach assumed missingness at random (MAR) and retained participants with partially observed outcome trajectories, foregoing imputation of missing outcome values. Modeling was performed using the nlme package (v3.1) in R.

## 4 Results

### 4.1 Participant retention

A total of *n* = 1058 users were assessed for eligibility, of which 744 were excluded and 314 completed the consent form. The flow of participant retention and reasons for exclusion are included in Fig. 1. Approximately 75.79% completed the baseline questionnaires (*n* = 238), 57.56% completed the one-month questionnaires (*n* = 137), and 41.18% completed the three-month questionnaires (*n* = 98). At baseline, 2.52% (*n* = 6) were excluded for failing the attention check question. At one month, 0.73% (*n* = 1) were excluded for failing to pass data integrity checks. At three months, no one was excluded for failing to pass data integrity checks. The final sample included *n* = 232 at baseline, *n* = 136 at one month, and *n* = 98 at three months. Our analyses showed that participants who completed the one-month follow-up questionnaires did not differ from participants who did not complete one-month follow-up questionnaires in their demographic characteristics, ACE exposure, baseline scores on the primary outcome, or baseline scores on the secondary outcomes (all *p* > .05). Our analyses showed that participants who completed the three-month follow-up questionnaires did not differ from participants who did not complete three-month follow-up questionnaires in their demographic characteristics, baseline scores on the primary outcome, or baseline scores on the secondary outcome (all *p* > .05). Participants who completed the three-month follow-up questionnaire reported a significantly lower number of ACEs at baseline (*M* = 2.02, *SD* = 2.01) compared to those who did not complete the three-month questionnaire (*M* = 3.05, *SD* = 2.34; *t*(187) = 3.49, *p* = .01).

**Fig. 1:**
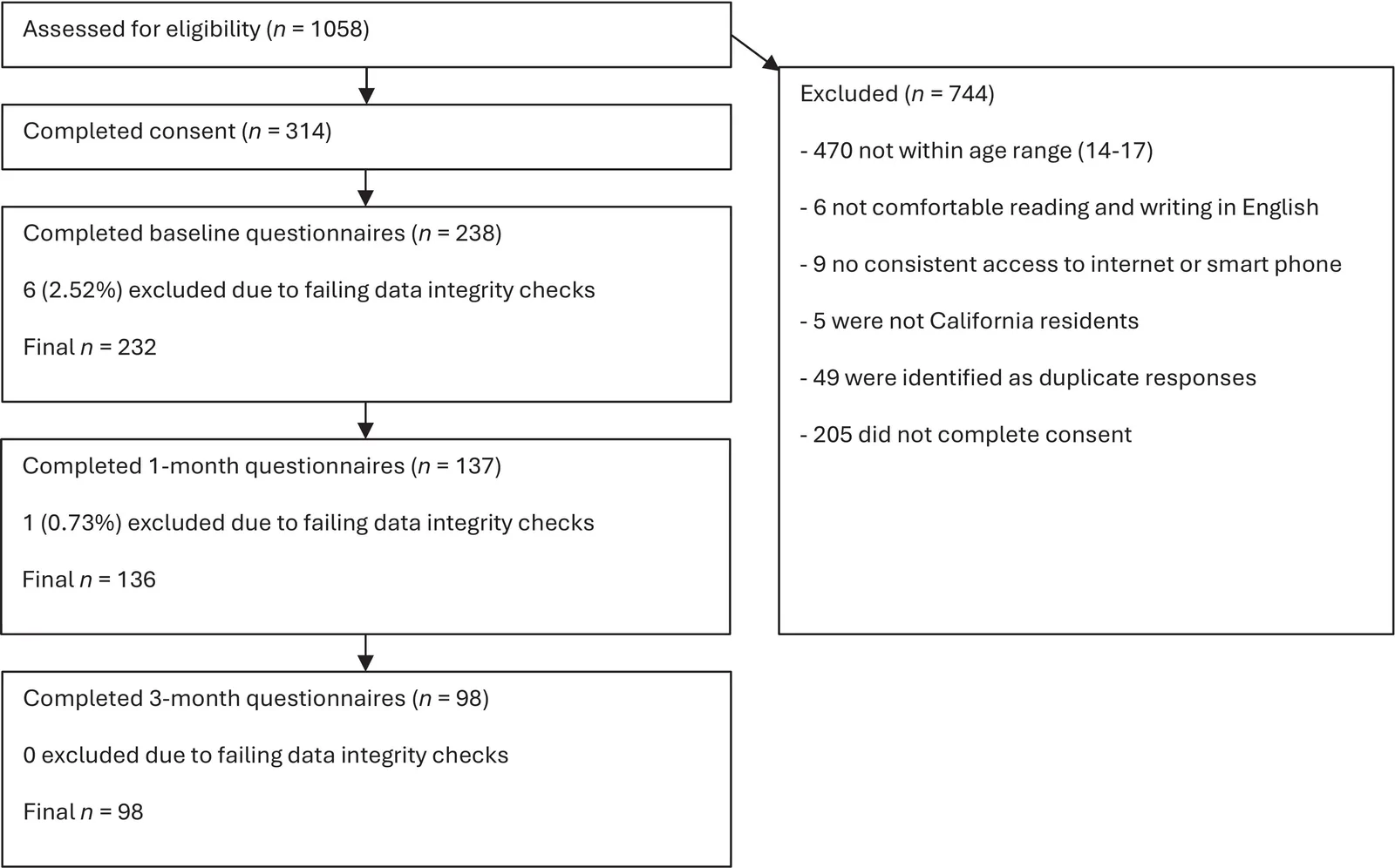
Participant retention Caption: The figure states that 1058 participants were assessed for eligibility. 744 were excluded. 470 were not within the age range (14–17), 6 were not comfortable reading and writing in English. 9 had no consistent access to internet or smart phone. 5 were not California residents. 49 were identified as duplicate responses. 205 did not complete consent. Of the 314 who completed the consent, 238 completed the baseline questionnaires, but 6 were excluded due to failing data integrity checks, so the final n was 232. 137 completed the one-month questionnaires, but 1 was excluded due to failing data integrity checks, so the final n was 136. 98 completed the three-month questionnaires, and zero were excluded due to failing data integrity checks.

### 4.2 Demographics

The sample included a majority of participants who were female assigned at birth (78.88%). Approximately 23.48% of participants were Transgender or Gender Diverse (defined as choosing any gender that was not exclusively “man/boy” or exclusively “woman/girl”) and 57.33% identified as LGBTQ+ (defined as choosing any sexual orientation that was not “heterosexual/straight”). The most common racial/ethnic identity was non-Hispanic White/Caucasian (46.98%) followed by Hispanic/Latinx (38.79%). Table 1 includes descriptive statistics for participants' demographic characteristics at baseline. For participant privacy, any categories with less than eleven participants were combined or included as “other.”

**Table 1:** Demographic characteristics at baseline.

	*N*	*%*
*Sex assigned at birth*		
Female	183	78.88
Male	47	20.26
*Gender Identity*⁎		
Man/Boy	54	23.28
Woman/Girl	145	62.5
Transgender	12	5.17
Trans man/masculine	18	7.76
Nonbinary	11	4.74
Unsure/questioning	14	6.03
Other	35	15.09
*Sexual Orientation*		
Heterosexual/Straight	99	42.67
Gay/Lesbian/Homosexual	18	7.76
Bisexual	49	21.12
Pansexual	11	4.74
Unsure/questioning	25	10.78
Other	30	12.93
*Race/Ethnicity*⁎		
American Indian or Alaska Native	11	4.74
Asian (including Asian Desi)	34	14.66
Black/African American	31	13.36
Hispanic/Latinx	90	38.79
White/Caucasian (non-Hispanic; includes Middle Eastern)	109	46.98
Other	15	6.46
Multiracial⁎⁎	49	21.97
*Grade*		
Seventh or Eighth	26	11.2
Ninth	44	18.97
Tenth	59	25.43
Eleventh	62	26.72
Twelfth	30	12.93
Freshman in college	11	4.74
*Adverse Childhood Experiences*		
Zero	42	18.42
One	44	19.29
Two or three	66	28.95
Greater than or equal to four	76	33.33

⁎ Participants permitted to select multiple options.

⁎⁎ Defined as choosing more than one race/ethnicity.

### 4.3 Risk detection

At baseline, 60.34% (*n* = 140) of participants triggered a risk response, by endorsing a non-zero response to any item on the SIDAS scale (besides one question that was reverse coded, in which case a non-ten response was indicative of risk), or a “yes” answer to the self-harm question, or a non-zero answer to item 4 of the YP-CORE (“I've thought of hurting myself”). Of the participants who triggered the risk response at baseline, 43 were not able to be reached after two phone calls and 30 indicated via text or email that they believed the risk flag was an error and they were not at risk of harming themselves. Among the 67 participants that were able to be contacted for a risk assessment, 51 indicated that they were fully capable of keeping themselves safe and were therefore provided with a list of mental health resources and 16 were determined to not to be at imminent risk of harm but could benefit from additional support, therefore a safety plan was created which was then emailed to the participant. At one month, 36.76% (*n* = 50) of participants triggered a risk response. Of these participants, 19 were not able to be reached, 11 indicated it was an error, 15 received a list of resources, and 5 created a safety plan. At three months, 29.59% (*n* = 29) of participants triggered a risk response. Of these participants, 9 were not able to be reached, 11 indicated it was an error, and 9 received a list of resources. Across all timepoints, no participants were identified as being at imminent risk, and as a result, no parental or emergency service contact was required.

### 4.4 Hypothesis 1a: Primary outcome

Among participants who completed the baseline and one-month questionnaires, a paired Wilcoxon signed rank test with continuity correction showed statistically significant within-person decreases in overall distress (YP-CORE) from baseline (*N* = 124, *M* = 21.29, *SD* = 6.89) to one-month (*N* = 124, *M* = 16.5, *SD* = 6.94; *V* = 5963, *p* < .001), with a large effect size (*r* = 0.62). Among participants who completed all three questionnaires, a repeated-measures ANOVA showed that distress was significantly different at the different time points, *F*(2, 154) = 22.32, *p*[GG] < 0.001, eta2[g] = 0.09. Post-hoc analyses with a Bonferroni adjustment revealed that there was a significant difference between baseline to one month, *t*(77) = 5.76, *p* < .001, *d* = 0.65, and baseline to three months, *t*(77) = 5.53, *p* < .001, *d* = 0.63, but not one month to three months *t*(77) = 1.26, *p* = .63.

Among participants who completed both the baseline and 1-month YP-CORE (*n* = 124), 33.06% (*n* = 41) saw a reliable decrease in distress (scores decreased by 7.9 or more), 4.84% (*n* = 6) saw a reliable increase in distress (scores increased by 7.9 or more), and 62.09% (*n* = 77) did not see reliable changes (changes in scores were < 7.9 and > −7.9). Among participants who completed both the baseline and 3-month YP-CORE *(n* = 81)*,* 34.57% (*n* = 28) saw a reliable decrease in distress, 1.23% (*n* = 1) saw a reliable increase in distress, and 64.19% (*n* = 52) did not see reliable changes. Table 2, Table 3, Table 4 present means and standard deviations for all outcomes at baseline, one-month, and three-months for all participants. Supplementary materials include descriptive statistics for participants who completed both baseline and one-month questionnaires (Table S1), as well as participants who completed all three questionnaires (Table S2).

**Table 2:** Descriptive statistics for distress, depression, anxiety, hopelessness, loneliness, suicidal ideation, perceived social support, attitudes about mental illness, expectancies, & quality of life across all time points.

	*N*	*Mean*	*SD*
*Distress (YP-CORE)*			
Baseline	227	21.51	7.11
One-month⁎	129	16.57	7.11
Three-months⁎⁎	98	16.33	7.51
*Depression (PHQ-8)*			
Baseline	226	12.06	5.66
One-month⁎	133	8.47	5.76
Three-months⁎⁎	97	8.80	5.79
*Anxiety (GAD-7)*			
Baseline	229	10.96	5.45
One-month⁎	135	7.66	5.45
Three-months⁎⁎	97	7.80	5.69
*Hopelessness (BHS-4)*			
Baseline	229	5.22	3.36
One-month⁎	135	3.69	3.15
Three-months⁎⁎	98	3.56	2.99
*Loneliness (ULS-3)*			
Baseline	230	6.75	1.83
One-month⁎	136	6.24	1.73
Three-months⁎⁎	98	5.93	1.93
*Suicidal Ideation (SIDAS)*			
Baseline	217	8.89	12.97
One-month⁎	133	3.75	9.09
Three-months⁎⁎	94	3.44	9.13
*Perceived Social Support (MSPSS): Friends*			
Baseline	231	18.22	5.93
One-month	136	18.77	5.87
Three-months	96	18.92	5.93
*Perceived Social Support (MSPSS): Family*			
Baseline	232	16.27	6.38
One-month	135	16.90	6.09
Three-months	97	17.15	5.51
*Attitudes about Mental Illness (AMIS): Negative Stereotypes*			
Baseline	231	11.04	3.62
One-month	136	11.06	3.67
Three-months	98	10.76	3.68
*Attitudes about Mental Illness (AMIS): Recovery and Outcomes*			
Baseline	232	20.61	3.08
One-month	136	20.57	3.16
Three-months	96	20.30	3.11
*Expectancies*			
Baseline	232	7.29	2.12
One-month	136	7.38	2.25
Three-months	98	6.81	2.47
*Physical-Health-Related Quality of Life (PROMIS) T-scores*†			
Baseline	230	46.63	7.64
One-month⁎	136	48.5	7.66
Three-months⁎⁎	97	49.38	8.76
*Mental-Health-Related Quality of Life (PROMIS) T-scores*†			
Baseline	232	40.13	7.73
One-month⁎	136	45.40	7.66
Three-months⁎⁎	97	45.81	7.69

⁎ Results indicate statistically-significant change from baseline to one-month.

⁎⁎ Results indicate statistically-significant change from baseline to three-months.

† *Raw summed scores are converted to T-scores (*Hays et al., 2009*; Intro to PROMIS*, 2021*). The general population mean is 50 with a standard deviation of 10.*

**Table 3:** Descriptive statistics for PROMIS Physical Health⁎ and Mental Health⁎ subscale T-scores across all time points.

	*Poor (*≤ *36); N (%)*	*Fair (>36 & ≤ 43); N (%)*	*Good (>43 & ≤ 51); N (%)*	*Very good (>51 & ≤ 58); N (%)*	*Excellent (>58); N (%)*
*Physical-Health-Related Quality of Life*					
Baseline	21 (9.13)	49 (21.30)	114 (49.57)	35 (15.22)	11 (4.78)
One-month	5 (3.68)	31 (22.79)	58 (42.65)	31 (22.79)	11 (8.09)
Three-months	7 (7.22)	14 (14.43)	45 (46.39)	19 (19.59)	12 (12.37)
	*Poor (≤34); N (%)*	*Fair (>34 & ≤ 42); N (%)*	*Good (>42 & ≤ 49); N (%)*	*Very good (>49 & ≤ 57; N (%)*	*Excellent (>57); N (%)*
*Mental- Health-Related Quality of Life*					
Baseline	55 (23.71)	90 (38.79)	61 (26.29)	24 (10.34)	2 (0.86)
One-month	14 (10.29)	32 (23.53)	49 (36.03)	35 (25.74)	6 (4.41)
Three-months	7 (7.22)	20 (20.62)	48 (49.48)	15 (15.46)	7 (7.22)

⁎ Raw summed scores are converted to T-scores and categorized according to T-score cut points (Hays et al., 2009; Intro to PROMIS, 2021). The general population mean is 50 with a standard deviation of 10.

**Table 4:** Descriptive statistics for questions related to self-harm, bullying, multisession services, and Soluna use across all time points.

	N total	N (%) positive	N (%) negative
*Self-harm question*			
Baseline	229	27 (11.79)	202 (88.21)
One-month	135	7 (5.19)	128 (94.81)
Three-months	97	7 (7.22)	90 (92.78)
*Bullying*			
Baseline	231	162 (70.13)	69 (29.87)
One-month	136	77 (56.62)	59 (43.38)
Three-months	98	54 (55.10)	44 (44.89)
*Multisession services: Overall*			
One-month	135	48 (35.56)	87 (64.44)
Three-months	98	38 (38.78)	60 (61.22)
*Multisession services: Within Soluna*			
One-month	48	18 (37.50)	30 (62.50)
Three-months	38	11 (28.95)	27 (71.05)

### 4.5 Hypothesis 1b: Secondary outcomes

Among participants who completed both the baseline and one-month questionnaires, there were statistically significant within-person decreases in depression (*V* = 5989.5, *p* < .001, *r* = 0.61), anxiety (*V* = 5812.5, *p* < .001, *r* = 0.51), hopelessness (*V* = 4334.5, *p* < .001, *r* = 0.47), loneliness (*V* = 3533.5, *p* < .001, *r* = 0.38), and suicidal ideation (*V* = 2159.5, *p* < .001, *r* = 0.52). There were statistically significant within-person increases in quality of life related to physical health (*V* = 1897.5, *p* = .008, *r* = 0.24) and mental health (*V* = 1174, *p* < .001, *r* = 0.56). There were no statistically significant differences in perceived social support, mental illness stigma, or expectancies. Additional details are available in Supplementary Materials (Tables S1-S2).

Among participants who completed all three questionnaires, depression, anxiety, hopelessness, loneliness, suicidal ideation, quality of life related to mental health, and quality of life related to physical health were significantly different at different time points (all *p* < .05). Similar to the primary outcome, post-hoc analyses suggested that depression, anxiety, hopelessness, loneliness, and suicidal ideation significantly decreased from baseline to one month and baseline to three months (all *p* < .05), but not one month to three-months (all *p* > .05). Quality of life related to mental health was significantly higher from baseline to one month and baseline to three months (*p* < .05), but not one month to three months (*p* > .05). Quality of life related to physical health was significantly higher from baseline to three months (*p* < .05), but was not different from baseline to one month or one month to three months (all *p* > .05). There were no statistically significant differences across the different time points in total difficulties, perceived social support, mental illness stigma, or expectancies. Additional details are available in Supplementary Materials (Tables S3-S4).

### 4.6 Hypothesis 2: Demographic and historical predictors

Results from multiple linear regressions showed that no sociodemographic or historical factors (including grade, gender identity, sex assigned at birth, sexual orientation, race/ethnicity, history of bullying, or number of ACEs) predicted changes in YP-CORE scores from baseline to one month. Table S3 in the Supplementary Materials includes detailed results. The results from a repeated-measures ANCOVA similarly found that no sociodemographic or historical factors predicted changes in YP-CORE scores across different time points. Table S4 in the Supplementary Materials includes detailed results.

### 4.7 Self-Reported Soluna use

Participants self-reported which features of Soluna they engaged in. Table 5 reports how many participants reported participating in each feature at the one-month and three-month follow-ups.

**Table 5:** Self-Reported Soluna use.

	One-Month N (%) 136	Three-Months N (%)
I've read, listened to, or watched content (e.g., a story or scenario)	75 (55.15)	70 (71.43)
I've used a tool (e.g., free write, mood log, or thought shaker)	94 (69.11)	72 (73.47)
I've responded or posted in the community space (e.g., a poll, forum, or live event)	39 (28.68)	45 (45.92)
I've chatted with a coach	39 (28.68)	40 (40.82)
I've made use of the emergency or crisis services	4 (2.94)	4 (4.08)
I've made use of the directory or additional services (e.g., food or housing)	4 (2.94)	10 (10.20)
None yet, I've only had a look around	27 (19.85)	8 (8.16)

### 4.8 Exploratory analysis

#### 4.8.1 Only users with elevated distress at baseline

A total of 184 users (79.31%) were classified as having elevated distress at baseline (defined as ≥14.1 on the YP-CORE; Twigg et al., 2016). Among these users, a wilcoxon signed rank test showed statistically significant within-person decreases in overall distress (YP-CORE) from baseline (*N* = 101, *M* = 23.67, *SD* = 5.11) to one-month (*N* = 101, *M* = 17.59, *SD* = 6.81; *V* = 5963, *p* < .001), with a large effect size (*r* = 0.76). A Friedman test showed that scores for distress were significantly different at the different time points, *X*2(2) = 41.0, *p* < .001. Post hoc analyses revealed that there was a significant difference between baseline to one month, *V* = 1774, *p* < .001, *r* = 0.75, and baseline to three months *V* = 1672, *p* < .001, *r* = 0.69, but not one month to three months, *V* = 858, *p* = .99.

#### 4.8.2 Sensitivity analysis

The results from independent mixed models for repeated measures were comparable to results obtained from pre-registered analyses. Detailed results are available in Tables S7-S9 in the supplementary materials.

### 4.9 Qualitative results

At the one-month timepoint, a total of 150 participants responded to the question, “*What are some things you liked about Soluna?*” Participants most frequently mentioned the ability to connect with coaches (*N* = 33, 22%) and the community/forum (*N* = 28, 18.67%). For example, one participant wrote, “*I really appreciate how the coaches truly take their time and are e1tremely [sic] personable!*” Another participant said, “*I like that [I] can read others post[s] and see that I'm not the only person going through my issues. That there others [sic] that feel the same way*.” A total of 143 participants responded to the question, “*What are some things you would change about Soluna?*” at the one-month timepoint. Participants commonly reported that they wanted additional content or tools (*N* = 32; 22.38%). One participant wrote that they would want “*more stories and tips, and more activities*.” Additionally, participants offered suggestions about changing some aspects of the design/user interface (*N* = 13, 9.09%), such as “*add a typing indicator to when you are chatting with a coach*.” Forty out of forty-seven participants who responded at the one-month timepoint to the question, “*Anything else you would like to share about your experience of Soluna?*” provided positive feedback. For example, one participant wrote, “*If someone is looking for mental health services, Soluna is something I would 1000% recommend.”*

## 5 Discussion

High rates of mental health concerns among youth in the US warrant the widespread deployment of innovative and scalable options for support. Soluna offers self-guided digital content, opportunities for peer community, and one-at-a-time, Single-Session-Therapy informed coaching sessions for free to all California youth aged 13 to 25 years old as part of California's CYBHI. In this pragmatic study, we examined mental health outcomes among a subset of self-selected Soluna users aged 14–17 over a three-month period. The results partially supported Hypothesis 1a: youth who completed follow-up questionnaires showed statistically-significant within-person decreases in the primary outcome of overall distress from baseline to one month. They also showed statistically-significant within-person decreases in overall distress from baseline to three months. However, there were no statistically significant changes from one-month to three months. Hypothesis 1b was partially supported. Significant within-person improvements across the three-month period were seen for the secondary outcomes of depression, anxiety, hopelessness, loneliness, quality of life, and suicidal ideation, but not perceived social support, outcome expectancies, or stigma.

We found the largest effect sizes for the primary outcome of overall distress (baseline to three months *d* = 0.63) and the secondary outcome of depression (baseline to three months *r* = 0.54). The results of this trial may be contextualized by comparing studies of other digital mental health programs. For example, in another single-arm, longitudinal study of an app-based mental health program, Alongside, analyses showed no significant differences in overall distress (YP-CORE) or depression (PHQ) scores from baseline to the 3-month follow-up (Cohen et al., 2025). Soluna is comparable to Alongside as a universal digital mental health and wellbeing platform delivered at scale to a youth population. Both platforms offer self-guided content and activities, but a key difference is that Soluna also offers opportunities to connect with others through community forums and behavioral health coaching. This aligns with literature suggesting that adding human elements to digital mental health programs may enhance their acceptability and effectiveness (Borghouts et al., 2021; Linardon et al., 2019). The Moderated Online Social Therapy (MOST) program is comparable to Soluna in several ways, as it offers self-guided tools, moderated peer community, and clinician-delivered web chats. Trials of MOST in adolescents have reported reductions in depression and anxiety among users over time (Alvarez-Jimenez et al., 2025). Our results add further evidence suggesting the potential utility of multi-component programs integrating multiple forms of support (i.e., self-guided, moderated peer support, human support) in a digital platform.

While there were statistically-significant decreases in distress from baseline to one-month and baseline to three-months, there were not statistically-significant decreases from one month to three months. The same pattern was seen for several secondary outcomes. We were unable to link participants in this study with their objective usage data, so it could not be determined whether the changes in outcomes over time were linked to engagement intensity. For example, it is possible that Soluna users in this study reported benefits up to three-months post-baseline because they continued to use the program over time. They may have derived benefit during their initial engagement with Soluna, with these early improvements being sustained rather than accumulative over time. It is also possible that users in this study did not see improvements between one-month and three-months because they discontinued their engagement. While participants answered one question regarding their self-reported usage of different features within Soluna, the question did not ask when or how often users engaged in these features, limiting our ability to determine whether or how usage patterns influenced changes in outcomes. It is also possible that users may have learned something new while initially engaging in the program that had a long-lasting impact. Research suggests that sudden, meaningful changes or “aha moments” can inspire “upward spirals” for continued improvement (Kaveladze et al., 2025; Wucherpfennig et al., 2017). Future research may more closely investigate the relationship between objective Soluna usage and psychosocial outcomes to determine whether effects were due to sustained engagement, meaningful insights gained during initial platform usage that continued to positively impact users' mental health, or other factors. Additionally, future research may include follow-ups past three months to determine how long the changes in outcomes are sustained.

Contrary to Hypothesis 1b, participants did not show significant increases in perceived social support, decreases in mental illness stigma, or increases in expectancies from baseline to either follow-up. There are several potential reasons why participants did not report improvements in these outcomes. Although Soluna encourages peer support through community forums, users are not permitted to directly contact each other for safety reasons. As a result, there were few opportunities to develop specific friendships with others, likely contributing to scores for perceived social support from friends remaining stable over time. Soluna may not be well-suited to target perceived social support from family, considering that the platform is targeted to individuals rather than family units. Additionally, because we were unable to link participants to their objective user data, it is unclear to what extent users in this study engaged in the community forums (e.g., number of comments posted). In previous studies of moderated peer support platforms, researchers found a correlation between participants' self-reported feelings of social connection and the number of comments they posted in the peer community (Alvarez-Jimenez et al., 2020). Future research should link objective user data to psychosocial outcomes to examine whether there are relationships between the degree program usage and changes on psychosocial outcomes, in addition to whether there are component-specific pathways to change.

Results suggest that participants entered the study with generally positive treatment expectancies and low levels of stigma. There may have been a ceiling/floor effect for these outcomes, meaning there was little room for change. Additionally, the internal consistencies for the AMIS subscales were low, raising concerns about interpretation. There is increasing research showing that acceptance and awareness of mental health among younger generations present differently than among older generations (Clarkin et al., 2024). It is possible that the questions used in this study to examine treatment expectancies and mental illness stigma no longer capture the experiences of younger people; alternative scales may need to be developed for younger generations.

Hypothesis 2 was supported; there was no evidence that sociodemographic or historical factors predicted within-person changes in distress among retained participants. These results align with other literature showing that demographic factors are not significant predictors of psychosocial outcomes in brief digital mental health programs (McDanal et al., 2024). Taken together, these findings may support the suggestion that digital mental health programs can provide equitable support to youth from diverse backgrounds (Schueller et al., 2019). At the same time, it is possible that differences between participants from varying backgrounds were of small magnitude and therefore not detectable in the present study.

Exploratory analyses found that effect sizes for distress were larger among users with elevated distress at baseline compared to the full sample (baseline to three months d = 0.69). This mirrors previous research on digital mental health programs that show larger effects among a clinically-elevated subsample of a larger population (Cohen et al., 2025; Osborn et al., 2020). Indeed, a large proportion of the sample was classified as having elevated distress at baseline (79.31%) and roughly one-third of participants reported having experienced four or more adverse childhood experiences. Research conducted in 2023 showed that 18.5% of high schoolers in the general population experienced four or more adverse childhood experiences (Swedo et al., 2024), suggesting that participants in this study had higher levels of trauma exposure compared to the general population. High levels of distress at baseline and high levels of trauma exposure among participants could indicate that these youth are more likely to seek out support through platforms such as Soluna than those experiencing low levels of distress or trauma. While Soluna is designed to be a universal wellbeing platform for all youth, these results highlight the importance of Soluna's built-in onramps for targeted support based on individual needs (e.g., coaching sessions, care coordination, etc.).

### 5.1 Strengths and limitations of the current study

The major strengths of the current study are that it was conducted by a team of researchers at an academic institution in collaboration with Kooth Digital Health, and that data was collected during real-world implementation of the Soluna platform. However, there are several limitations. First, the present sample is not representative of all youth who engage in the Soluna platform. We chose to focus on youth aged 14–17, but Soluna is available to all youth aged 13–25, limiting our ability to draw conclusions about youth aged 13 or older than 17. Youth who were willing to complete multiple questionnaires within the study may represent a highly engaged or motivated subset of users. A limitation of this trial is that we are unable to determine whether these results would generalize to youth who did not wish to take part in a formal, paid research study. Additionally, the sample includes a smaller proportion of youth who identified as Hispanic/Latinx than would be expected from population estimates. In the current sample, 38.79% of participants identified as Hispanic/Latinx, while previous research shows that approximately 51.9% of Californians under 17 identify as Hispanic (Johnson et al., 2025). This may be due to inclusion criteria that required English proficiency and/or hesitation to participate in research given historical factors and the current political context surrounding immigration law enforcement (Rojo et al., 2024).

Another limitation of the current study is the lack of a control group to determine if the changes in outcomes over time were due to factors outside of using the Soluna platform. As the platform was available for free to all youth in California, a randomized controlled trial was not possible or ethical to perform. As a result, direct claims regarding causality cannot be made. It is possible that the results from this longitudinal study reflect regression to the mean rather than a true reduction in distress that is attributable to the Soluna platform. Participants may have been experiencing an unusually large amount of psychological distress when they started engaging with Soluna and may have returned to their more average amount of psychological distress at the follow-up timepoints. It is also possible that outside factors could have contributed to the changes in psychosocial outcomes beyond engagement in the Soluna platform. For example, participants may have received mental health services alongside Soluna within the study period. These limitations are inherent in any single-arm naturalistic study in which it is impossible to randomize participants. At the same time, these limitations necessitate cautious interpretation of results, as changes in outcomes cannot be definitively attributed to Soluna. It is also important to note that we focused on statistically-significant change, as opposed to pre-specifying meaningful clinical change; this is a function of our pre-registration. We included the reliable change index as an additional analysis to supplement our findings. Future research may demonstrate potential effects more clearly by pre-specifying benchmarks for clinically-meaningful change.

We used only complete cases in our pre-registered analyses. We conducted sensitivity analyses utilizing all available outcome observations and found comparable results to pre-registered analyses. However, our findings are limited by lack of available follow-up data for all participants. It is possible, for example, that users who felt more positively about Soluna were more likely to continue filling out surveys related to Soluna. Our analyses showed that participants who completed the follow-up questionnaires did not differ from participants who did not complete follow-up questionnaires in terms of their demographics or baseline distress, but participants who completed the three-month follow-up questionnaire reported a significantly lower number of ACEs at baseline on average compared to those who did not complete the three-month questionnaire. This could suggest that reductions in distress seen at three months were due to the inclusion of fewer participants with high trauma exposure. However, there was no significant difference in ACE exposure on average between participants who completed the one-month questionnaire and those who did not, suggesting that significant results at the one-month follow-up may not be attributed to differences in baseline ACE exposure.

We were unable to link participants in this study with their objective usage data (e.g., logins, days active, time using specific tools, etc.), limiting our ability to examine potential relationships between improvements in psychosocial outcomes with the degree or type of program usage. Because researchers at Anonymous Institution collected data from participants on an independent Qualtrics survey outside of the Soluna platform, in a de-identified manner, objective user engagement data could not be linked to specific participants. Although we asked for self-reported use, there are significant limitations to the accuracy or utility of this data point (e.g., recall bias, lack of specific information on number of interactions, duration, quality, or timing). Future research should link objective user data to psychosocial outcomes to examine component-specific pathways to change.

The majority of participants in this study were assigned female at birth. This aligns with research indicating that people assigned female at birth are more likely to seek mental health services (Gonzalez et al., 2011). Additionally, the majority of participants in this study identified within the LGBTQ+ community. This is consistent with previous studies on digital, self-administered mental health programs (Schleider et al., 2022). It is possible that LGBTQ+ youth are particularly interested in digital, self-administered programs because of the barriers that LGBTQ+ youth face in finding accessible and affirming providers (Szkody et al., 2025). In testing Hypothesis 2, we found no evidence that sex assigned at birth, gender identity, or sexual orientation predicted changes in distress. However, conducting future research with a larger sample size may be useful to delineate any significant differences that may not have been detected in the current study.

In an effort to understand participants' use of mental health services both within and outside of Soluna, we asked participants at follow-up assessments whether they saw a mental health professional more than once, and whether this was through Soluna or outside of Soluna. However, this question was limited in its ability to fully characterize participants' use of mental health services, as it was inclusive of Soluna as well as other external services. Future research should more clearly distinguish support received from Soluna and support received externally. Additionally, it is important to measure the frequency at which participants seek external support and the type of support they receive, including medication, school counseling, therapy, crisis support, and other concurrent care.

The research team at Northwestern University employed strict crisis procedures within the surveys to ensure the safety of all participants in the research study, in addition to Soluna's standard ongoing risk and safeguarding procedures. However, many participants indicated that they were flagged incorrectly by the surveys and were not at risk of harming themselves, suggesting that the research team's crisis procedures may have been overly sensitive. This points to the importance of understanding and capturing the nuances between passive suicidal ideation and active suicidal ideation. In the future, researchers may consider how to measure active suicidal ideation and only follow-up with participants who are at imminent risk, while also ensuring they are meeting ethical standards. Additionally, youth in this study who indicated risk on the surveys received extra outreach through the research team's crisis procedures. A limitation to this study is that it is not possible to differentiate whether the research team's crisis procedures conferred any additional effects on top of those effects that may have been conferred by engagement in Soluna.

## 6 Conclusion

Overall, the current findings add to the increasing amount of evidence that digital mental health tools may serve as accessible, scalable supports for youth, across a broad demographic. Soluna users in this study experienced statistically-significant within-person reductions in the primary outcome of distress, as well as secondary outcomes depression, anxiety, hopelessness, loneliness, and suicidal ideation across a three-month period. Although the lack of a control group prohibits causal interpretation, the study's pragmatic design helps strengthen ecological validity and reflects the real-world implementation and conditions in which digital mental health tools are deployed broadly. These results highlight the potential of digital mental health platforms as viable tools to a public health approach to youth mental health. Continued research is needed to examine longer-term outcomes and link outcomes to objective usage data in order to determine the degree to which Soluna usage contributes to changes in outcomes. This study highlights the value of academic and industry partnerships, as well as the value state governments can provide to implement digital mental health and wellbeing resources across a large population.

## CRediT authorship contribution statement

**Katherine Cohen:** Conceptualization, Data curation, Formal analysis, Writing – original draft, Writing – review & editing. **Andy Rapoport:** Data curation, Project administration, Writing – review & editing. **Shannon Hill:** Data curation, Project administration, Writing – review & editing. **Regina Misch:** Conceptualization, Writing – review & editing. **Emily P. Cowling:** Conceptualization, Funding acquisition, Writing – review & editing. **Louisa Salhi:** Conceptualization, Funding acquisition, Writing – review & editing. **Jessica L. Schleider:** Conceptualization, Supervision, Writing – review & editing.

## Declaration of competing interest

The authors declare the following financial interests/personal relationships which may be considered as potential competing interests: JS reports financial support was provided by Kooth Digital Health Limited. JS reports a relationship with Walden Wise that includes: board membership. Jessica Schleider reports a relationship with Koko that includes: board membership. JS reports a relationship with United Health that includes: consulting or advisory. JS reports a relationship with Woebot that includes: consulting or advisory. JS reports a relationship with Navi that includes: board membership and equity or stocks. EC and LS are employees of Kooth Digital Health and receive honorarium and shares in Kooth annually; however, these are not contingent on study performance or scientific outputs. LS is also an honorary senior research fellow at the University of Manchester. RM was employed by Kooth Digital Health at the time of data collection for this study. RM is founder for Threshold LLC, which was established during the reviewing and editing phase of this manuscript. The authors do not receive any financial benefit from the outcomes of this study. If there are other authors, they declare that they have no known competing financial interests or personal relationships that could have appeared to influence the work reported in this paper.
