# Supplementary material for: Psychological distress over time among adolescent Soluna users: A prospective observational study
Source: Internet Interv. 2026 Sep 24;46:101001. doi: 10.1016/j.invent.2026.101001 (PMC13673023; doi:10.1016/j.invent.2026.101001)
Supplement: Supplementary file 1 [file mmc1.docx]

# Supplementary materials

| Table S1. Descriptive statistics for YP-CORE, PHQ-8, GAD-7, BHS-4, ULS-3, SIDAS, MSPSS, AMIS, Expectancies, & PROMIS among participants who completed both baseline and one-month questionnaires. | | | | |
| --- | --- | --- | --- | --- |
|  | *N* | *Mean* | *SD* | *Results* |
| *YP-CORE* |  |  |  |  |
| Baseline | 124 | 21.29 | 6.89 | Statistically significant decrease (V = 5963, p < .001), with a large effect size (r = 0.62) |
| One-month | 124 | 16.5 | 6.94 |  |
| *PHQ-8* |  |  |  |  |
| Baseline | 128 | 11.97 | 5.72 | Statistically significant decrease (V = 5989.5, p < .001), with a large effect size (r = 0.61) |
| One-month | 128 | 8.56 | 5.69 |  |
| *GAD-7* |  |  |  |  |
| Baseline | 131 | 10.56 | 5.31 | Statistically significant decrease (V = 5812.5, p < .001), with a large effect size (r = 0.51) |
| One-month | 131 | 7.67 | 5.41 |  |
| *BHS-4* |  |  |  |  |
| Baseline | 131 | 4.99 | 3.34 | Statistically significant decrease (V = 5812.5, p < .001), with a moderate effect size (r = 0.47) |
| One-month | 131 | 3.76 | 3.14 |  |
| *ULS-3* |  |  |  |  |
| Baseline | 131 | 6.80 | 1.82 | Statistically significant decrease (V = 3533.5, p < .001), with a moderate effect size (r = 0.38) |
| One-month | 131 | 6.22 | 1.74 |  |
| *SIDAS* |  |  |  |  |
| Baseline | 119 | 7.66 | 12.30 | Statistically significant decrease (V = 2159.5, p < .001), with a large effect size (r = 0.52) |
| One-month | 119 | 3.95 | 9.44 |  |
| *MSPSS: Friends* |  |  |  |  |
| Baseline | 131 | 18.23 | 5.32 | No statistically significant difference (V = 2632, p = 0.18). |
| One-month | 131 | 18.78 | 5.81 |  |
| *MSPSS: Family* |  |  |  |  |
| Baseline | 131 | 16.56 | 6.64 | No statistically significant difference (V = 2656.5, p = 0.49). |
| One-month | 131 | 16.95 | 5.95 |  |
| *AMIS: Negative Stereotypes* |  |  |  |  |
| Baseline | 131 | 10.85 | 3.59 | No statistically significant difference (V = 2705, p = 0.80). |
| One-month | 131 | 10.98 | 3.62 |  |
| *AMIS: Recovery and Outcomes* |  |  |  |  |
| Baseline | 132 | 20.63 | 3.15 | No statistically significant difference (V = 2493, p = 0.95). |
| One-month | 132 | 20.57 | 3.15 |  |
| *Expectancies* |  |  |  |  |
| Baseline | 132 | 7.47 | 2.04 | No statistically significant difference (V = 2667.5, p = 0.82). |
| One-month | 132 | 7.43 | 2.22 |  |
| *PROMIS: Physical-Health-Related Quality of Life** |  |  |  |  |
| Baseline | 132 | 46.6 | 7.82 | Statistically significant increase (V = 1897.5, p = .008), with a small effect size (r = 0.24) |
| One-month | 132 | 48.36 | 7.63 |  |
| *PROMIS: Mental-Health-Related Quality of Life** |  |  |  |  |
| Baseline | 132 | 40.75 | 7.58 | Statistically significant increase (V = 1174, p < .001), with a large effect size (r = 0.56) |
| One-month | 132 | 45.36 | 7.55 |  |

**Raw summed scores are converted to T-scores (Hays et al., 2009; Intro to PROMIS, 2021)*

| Table S2. Descriptive statistics for YP-CORE, PHQ-8, GAD-7, BHS-4, ULS-3, SIDAS, MSPSS, AMIS, Expectancies, & PROMIS among participants who completed all three questionnaires. | | | | |
| --- | --- | --- | --- | --- |
|  | *N* | *Mean* | *SD* | *Results* |
| *YP-CORE* |  |  |  |  |
| Baseline | 78 | 20.9 | 6.74 | A repeated-measures ANOVA showed that scores for distress were significantly different at the different time points, F(2, 154) = 22.32, p[GG] < .001, eta2[g] = 0.09. Post-hoc analyses with a Bonferroni adjustment revealed that there was a significant difference between baseline to one month, t(77) = 5.76, p < .001, d = 0.65, and baseline to three months, t(77) = 5.53, p < .001, d = 0.63, but not one month to three months t(77) = 1.26, p = .63.  There was a 22.97% decrease in mean YP-CORE scores from baseline to three-months. |
| One-month | 78 | 17.0 | 6.58 |  |
| Three-months | 78 | 16.1 | 7.13 |  |
| *PHQ-8* |  |  |  |  |
| Baseline | 78 | 11.6 | 5.66 | A Friedman test showed that scores for depression were significantly different at the different time points, X2(2) = 28.03, p < .001. Post hoc analyses revealed that there was a significant difference between baseline to one month, V = 2102, p < .001, r = 0.54, and baseline to three months V = 2083, p < .001, r = 0.53 but not one month to three months, V = 1331, p = .99.  There was a 26.38% decrease in mean PHQ scores from baseline to three-months. |
| One-month | 78 | 8.59 | 5.62 |  |
| Three-months | 78 | 8.54 | 5.62 |  |
| *GAD-7* |  |  |  |  |
| Baseline | 81 | 10.2 | 5.07 | A Friedman test showed that scores for anxiety were significantly different at the different time points, X2(2) = 19.95, p < .001. Post hoc analyses revealed that there was a significant difference between baseline to one month, V = 1952, p < .001, r = 0.41, and baseline to three months V = 1915, p < .001, r = 0.45, but not one month to three months, V = 1514, p = .79.  There was a 24.80% decrease in mean GAD scores from baseline to three-months. |
| One-month | 81 | 8.2 | 5.30 |  |
| Three-months | 81 | 7.67 | 5.26 |  |
| *BHS-4* |  |  |  |  |
| Baseline | 81 | 4.74 | 3.36 | A Friedman test showed that scores for hopelessness were significantly different at the different time points, X2(2) = 21.39, p < .001. Post hoc analyses revealed that there was a significant difference between baseline to one month, V = 1596, p = .005, r = 0.35, and baseline to three months V = 1564, p < .001, r = 0.48, but not one month to three months, V = 1062, p = .32.  There was a 28.06% decrease in mean BHS scores from baseline to three-months. |
| One-month | 81 | 3.79 | 3.14 |  |
| Three-months | 81 | 3.41 | 3.00 |  |
| *ULS-3* |  |  |  |  |
| Baseline | 82 | 6.77 | 1.93 | A Friedman test showed that scores for loneliness were significantly different at the different time points, X2(2) = 21.85, p < .001. Post hoc analyses revealed that there was a significant difference between baseline to one month, V = 1303, p = .01, r = 0.33, and baseline to three months V = 1342, p < .001, r = 0.48, but not one month to three months, V = 840, p = .06.  There was a 13.74% decrease in mean ULS scores from baseline to three-months. |
| One-month | 82 | 6.27 | 1.78 |  |
| Three-months | 82 | 5.84 | 1.93 |  |
| *SIDAS* |  |  |  |  |
| Baseline | 70 | 7.46 | 12.3 | A Friedman test showed that scores for suicidal ideation were significantly different at the different time points, X2(2) = 21.79, p < .001. Post hoc analyses revealed that there was a significant difference between baseline to one month, V = 745, p < .001, r = 0.49, and baseline to three months V = 619, p = .004, r = 0.41, but not one month to three months, V = 144, p = .99.  There was a 53.62% decrease in mean SIDAS scores from baseline to three-months. |
| One-month | 70 | 3.83 | 9.37 |  |
| Three-months | 70 | 3.46 | 8.96 |  |
| *MSPSS: Friends* |  |  |  |  |
| Baseline | 81 | 18.9 | 4.99 | A Friedman test showed that scores for social support from friends were not significantly different at the different time points, X2(2) = 2.86, p = .31. |
| One-month | 81 | 19.2 | 5.42 |  |
| Three-months | 81 | 19.2 | 5.9 |  |
| *MSPSS: Family* |  |  |  |  |
| Baseline | 80 | 16.7 | 6.61 | A repeated-measures ANOVA showed that scores for social support from family were not significantly different at the different time points, F(2, 158) = 2.09, p[GG] =.19. |
| One-month | 80 | 17.2 | 6.12 |  |
| Three-months | 80 | 17.8 | 5.32 |  |
| *AMIS: Negative Stereotypes* |  |  |  |  |
| Baseline | 81 | 10.7 | 3.67 | A Friedman test showed that scores for negative stereotypes were not significantly different at the different time points, X2(2) = 0.14, p = .93. |
| One-month | 81 | 11.0 | 3.58 |  |
| Three-months | 81 | 10.7 | 3.66 |  |
| *AMIS: Recovery and Outcomes* |  |  |  |  |
| Baseline | 81 | 20.9 | 2.78 | A Friedman test showed that scores for recovery and outcomes were not significantly different at the different time points, X2(2) = 1.29, p = .57. |
| One-month | 81 | 20.6 | 2.97 |  |
| Three-months | 81 | 20.5 | 2.96 |  |
| *Expectancies* |  |  |  |  |
| Baseline | 82 | 7.4 | 2.1 | A Friedman test showed that scores for expectancies were not significantly different at the different time points, X2(2) = 2.41, p = .35. |
| One-month | 82 | 7.28 | 2.24 |  |
| Three-months | 82 | 6.90 | 2.43 |  |
| *PROMIS: Physical-Health-Related Quality of Life** |  |  |  |  |
| Baseline | 82 | 47.1 | 7.88 | A Friedman test showed that scores for quality of life related to physical health were significantly different at the different time points, X2(2) = 10.35, p = .009. Post hoc analyses revealed that there was a significant difference between baseline to three months, V = 708, p = .005, r = 0.35, but not baseline to one month V = 866, p = .99, or one month to three months, V = 822, p = .21.  There was a 5.31% increase in mean physical-health-related quality of life scores from baseline to three-months. |
| One-month | 82 | 48.0 | 7.25 |  |
| Three-months | 82 | 49.6 | 8.79 |  |
| *PROMIS: Mental-Health-Related Quality of Life** |  |  |  |  |
| Baseline | 81 | 41.3 | 7.64 | A Friedman test showed that scores for quality of life related to mental health were significantly different at the different time points, X2(2) = 24.84, p < .001. Post hoc analyses revealed that there was a significant difference between baseline to one month, V = 612, p < .001, r = 0.49, and baseline to three months V = 508, p < .001, r = 0.52, but not one month to three months, V = 1122, p = .63.  There was a 11.86% increase in mean mental-health-related quality of life scores from baseline to three-months. |
| One-month | 81 | 44.9 | 7.07 |  |
| Three-months | 81 | 46.2 | 7.51 |  |

**Raw summed scores are converted to T-scores (Hays et al., 2009; Intro to PROMIS, 2021)*

| Table S3: Hypothesis 2 Linear regression results at one month. | | | | |
| --- | --- | --- | --- | --- |
|  | Estimate | Std. Error | t-value | Pr(>\|t\|) |
| Intercept | 11.84 | 6.14 | 1.93 | .06 |
| Baseline YP-CORE | 0.45 | 0.10 | 4.29 | 4.82e-05 |
| Grade | -0.42 | 0.55 | -0.76 | .45 |
| Gender: TGD | -4.84 | 4.95 | -0.98 | .33 |
| Gender: Woman/Girl | -7.13 | 4.99 | -1.43 | .16 |
| Race/Ethnicity: Asian (including Asian Desi) | 0.79 | 2.21 | 0.36 | .72 |
| Race/Ethnicity: Black/African American | -1.98 | 2.28 | -0.87 | .39 |
| Race/Ethnicity: Hispanic/Latinx | -2.53 | 1.72 | -1.47 | .14 |
| Race/Ethnicity: Multiracial | -2.48 | 1.82 | -1.36 | .18 |
| Race/Ethnicity: Other | -3.90 | 5.89 | -0.66 | .51 |
| ACES | 0.23 | 0.32 | 0.70 | .48 |
| Bullying history | 2.64 | 1.51 | 1.75 | .08 |
| Sex: Female | 3.73 | 4.63 | 0.80 | .42 |
| Sexual orientation: Other | -2.0 | 4.37 | -0.46 | .65 |
| Sexual orientation: Asexual | 7.21 | 7.70 | 0.94 | .35 |
| Sexual orientation: Bisexual | 0.76 | 1.53 | 0.49 | .62 |
| Sexual orientation: Gay/Lesbian/Homosexual | -1.89 | 2.65 | -0.71 | .48 |
| Sexual orientation: No label | -3.45 | 4.52 | -0.76 | .45 |
| Sexual orientation: pansexual | -0.12 | 3.14 | -0.04 | .97 |
| Sexual orientation: Queer | -0.20 | 3.31 | -0.06 | .95 |
| Sexual orientation: Unsure/Questioning | 3.70 | 2.01 | 1.84 | .07 |
| *Overall results* | | | | |
| Residual standard error: 5.62 on 83 degrees of freedom | | | | |
| Adjusted R-squared: 0.30 | | | | |
| F-statistic: 3.26 on 20 and 83 DF, p-value < ,001 | | | | |

| Table S4. Hypothesis 2 ANCOVA results at three months. | | | | | | |
| --- | --- | --- | --- | --- | --- | --- |
|  | Df | Test stat | Approx F | Num Df | Den Df | Pr(>F) |
| Intercept | 1 | 0.08 | 2.24 | 2 | 46 | .12 |
| Baseline YP-CORE | 1 | 0.27 | 8.71 | 2 | 46 | .0006 |
| Grade | 1 | 0.02 | 0.49 | 2 | 46 | .61 |
| Gender | 2 | 0.18 | 2.34 | 4 | 94 | .06 |
| Race/Ethnicity | 41 | 0.24 | 1.59 | 8 | 94 | .14 |
| ACES | 1 | 0.008 | 0.18 | 2 | 46 | 0.83 |
| History of bullying | 1 | 0.06 | 1.57 | 2 | 46 | 0.22 |
| Sex | 1 | 0.05 | 1.31 | 2 | 46 | 0.28 |
| Sexual orientation | 8 | 0.29 | 1.00 | 16 | 94 | 0.46 |

| Table S5. Exploratory analyses linear regression results at one-month. | | | | |
| --- | --- | --- | --- | --- |
|  | Estimate | Std. Error | T value | Pr(>\|t\|) |
| Intercept | 5.23 | 1.98 | 2.63 | .009 |
| Baseline YP-CORE | 0.55 | 0.08 | 7.17 | 6.94e-11 |
| Peer-connected | -1.29 | 1.79 | -0.72 | .47 |
| Professional-supported | 0.67 | 1.56 | 0.43 | .67 |
| Self-directed | -1.32 | 1.51 | -0.87 | 0.38 |

**Strengths and Difficulties Questionnaire (SQQ)**

This measure includes 25 items that ask youth to indicate their strengths and difficulties (e.g., “I get very angry and often lose my temper”) by selecting either 0 = Not True, 1 = Somewhat True or 2 = Certainly True. There are five subscales including emotional problems, conduct problems, hyperactivity, peer problems, and prosocial, each including five items. The Total Difficulties Score is a sum of the first four subscales ranging from 0-40, with higher scores indicating greater difficulties (Goodman, 2001). The SDQ was completed at all three timepoints. Internal consistency for Total Difficulties was α = .45 at baseline, α = .53 at one month, and α = .44 at three-months. Internal consistency for the prosocial subscale was α = .66 at baseline, α = .56 at one month, and α = .64 at three-months. Our team elected to report the analyses using the SDQ in the supplementary materials rather than the manuscript due to low reliability.

| Table S6. Results pertaining to SDQ. | | | | |
| --- | --- | --- | --- | --- |
| *Participants who completed both baseline and one-month questionnaires.* | | | | |
|  | *N* | *Mean* | *SD* | *Results* |
| *Total Difficulties* |  |  |  |  |
| Baseline | 127 | 17.15 | 3.84 | Statistically-significant decrease (*t*(126) = 2.56, p =.01) with a small effect size (d = 0.17). |
| One-month | 127 | 16.44 | 4.37 |  |
| *Prosocial Subscale* |  |  |  |  |
| Baseline | 132 | 8.09 | 1.62 | No statistically significant difference (V = 1879.5, p = 0.49). |
| One-Month | 132 | 8.19 | 1.66 |  |
| *Participants who completed all three questionnaires.* | | | | |
|  | *N* | *Mean* | *SD* | *Results* |
| *Total Difficulties* |  |  |  |  |
| Baseline | 75 | 16.7 | 3.48 | A repeated-measures ANOVA showed that scores were not significantly different at the different time points, F(2, 148) = 2.99, p[GG] =.06. |
| One-month | 75 | 15.9 | 4.38 |  |
| Three-months | 75 | 15.7 | 3.97 |  |
| *Prosocial Subscale* |  |  |  |  |
| Baseline | 81 | 8.18 | 1.61 | A Friedman test showed that scores were not significantly different at the different time points, X2(2) = 1.01, p = .60. |
| One-Month | 81 | 8.32 | 1.51 |  |
| Three-months | 81 | 8.36 | 1.55 |  |

**Sensitivity Analysis**

As a sensitivity analysis, we ran 13 independent mixed models for repeated measures (MMRM; one per outcome) which utilized all available outcome observations. Time was modeled as a categorical variable with three levels (baseline, 1 month, 3 months). Within-participant covariance was represented using an unstructured covariance matrix (wherein each time point can have a different variance, and each pair of time points can have a different correlation), and model parameters were estimated using restricted maximum likelihood. Model-estimated marginal means and pairwise contrasts were used to evaluate change between time points. Bonferroni correction was employed within outcome contrasts to correct for multiple hypotheses. Importantly, this approach assumed missingness at random (MAR) and retained participants with partially observed outcome trajectories, foregoing imputation of missing outcome values. Modeling was performed using the *nlme* package (v3.1) in R.

**Table S7. Main Effect of Time on Each Outcome (Omnibus Test)**

| **Outcome** | **F-value** | **p-value** | **Significant** |
| --- | --- | --- | --- |
| Distress (YP-CORE) | 44.17 | < 0.0001 | Yes |
| Depression (PHQ) | 44.57 | < 0.0001 | Yes |
| Anxiety (GAD) | 29.58 | < 0.0001 | Yes |
| Hopelessness (BHS) | 26.95 | < 0.0001 | Yes |
| Stigma: Negative Stereotypes (AMIS) | 1.09 | 0.3382 | No |
| Stigma: Recovery and Outcomes (AMIS) | 0.48 | 0.619 | No |
| Loneliness (ULS) | 15.68 | < 0.0001 | Yes |
| Social Support: Friends (MSPSS) | 0.99 | 0.3728 | No |
| Social Support: Family (MSPSS) | 1.06 | 0.3462 | No |
| Expectancies | 1.70 | 0.184 | No |
| Global Physical Health (PROMIS) | 9.461 | 0.0001 | Yes |
| Global Mental Health (PROMIS) | 47.56 | < 0.0001 | Yes |
| Suicidal Ideation (SIDAS) | 20.93 | < 0.0001 | Yes |

**Table S8. Per Time Point Marginal Means for Each Outcome**

| **Outcome** | **Time** | **Estimate** | **Standard Error** | **DF** | **95% CI** | **p-value** |
| --- | --- | --- | --- | --- | --- | --- |
| Distress  (YP-CORE) | Baseline | 21.6 | 0.473 | 225 | [20.6 – 22.5] | < 0.0001 |
|  | 1 month | 16.8 | 0.585 | 156 | [15.6 – 17.9] | < 0.0001 |
|  | 3 months | 16.2 | 0.707 | 114 | [14.8 – 17.6] | < 0.0001 |
| Depression (PHQ) | Baseline | 12.05 | 0.374 | 232 | [11.31 – 12.79] | < 0.0001 |
|  | 1 month | 8.62 | 0.448 | 175 | [7.73 – 9.50] | < 0.0001 |
|  | 3 months | 8.88 | 0.502 | 134 | [7.89 – 9.88] | < 0.0001 |
| Anxiety (GAD) | Baseline | 10.98 | 0.360 | 231 | [10.3 – 11.69] | < 0.0001 |
|  | 1 month | 7.98 | 0.443 | 154 | [7.1 – 8.85] | < 0.0001 |
|  | 3 months | 7.91 | 0.509 | 124 | [6.9 – 8.92] | < 0.0001 |
| Hopelessness (BHS) | Baseline | 5.20 | 0.221 | 232 | [4.76 – 5.64] | < 0.0001 |
|  | 1 month | 3.86 | 0.243 | 175 | [3.38 – 4.34] | < 0.0001 |
|  | 3 months | 3.60 | 0.264 | 127 | [3.08 – 4.12] | < 0.0001 |
| Stigma: Negative Stereotypes (AMIS) | Baseline | 11.1 | 0.237 | 233 | [10.6 – 11.6] | < 0.0001 |
|  | 1 month | 11.2 | 0.265 | 201 | [10.7 – 11.7] | < 0.0001 |
|  | 3 months | 10.8 | 0.307 | 140 | [10.2 – 11.4] | < 0.0001 |
| Stigma: Recovery and Outcomes (AMIS) | Baseline | 20.6 | 0.202 | 234 | [20.2 – 21.0] | < 0.0001 |
|  | 1 month | 20.5 | 0.241 | 177 | [20.0 – 21.0] | < 0.0001 |
|  | 3 months | 20.4 | 0.275 | 136 | [19.8 – 20.9] | < 0.0001 |
| Loneliness (ULS) | Baseline | 6.76 | 0.120 | 232 | [6.52 – 7.00] | < 0.0001 |
|  | 1 month | 6.23 | 0.134 | 175 | [5.96 – 6.49] | < 0.0001 |
|  | 3 months | 5.89 | 0.172 | 119 | [5.55 – 6.23] | < 0.0001 |
| Social Support: Friends (MSPSS) | Baseline | 18.2 | 0.389 | 231 | [17.4 – 19.0] | < 0.0001 |
|  | 1 month | 18.7 | 0.465 | 169 | [17.8 – 19.6] | < 0.0001 |
|  | 3 months | 18.7 | 0.540 | 122 | [17.6 – 19.7] | < 0.0001 |
| Social Support: Family (MSPSS) | Baseline | 16.3 | 0.420 | 231 | [15.4 – 17.1] | < 0.0001 |
|  | 1 month | 16.6 | 0.442 | 198 | [15.7 – 17.5] | < 0.0001 |
|  | 3 months | 16.9 | 0.480 | 126 | [16.0 – 17.9] | < 0.0001 |
| Expectancies | Baseline | 7.29 | 0.145 | 234 | [7.00 – 7.57] | < 0.0001 |
|  | 1 month | 7.30 | 0.182 | 159 | [6.94 – 7.66] | < 0.0001 |
|  | 3 months | 6.91 | 0.230 | 116 | [6.45 – 7.36] | < 0.0001 |
| Global Physical Health (PROMIS) | Baseline | 46.7 | 0.502 | 231 | [45.7 – 47.7] | < 0.0001 |
|  | 1 month | 48.4 | 0.587 | 176 | [47.2 – 49.5] | < 0.0001 |
|  | 3 months | 49.2 | 0.747 | 140 | [47.8 – 50.7] | < 0.0001 |
| Global Mental Health (PROMIS) | Baseline | 40.1 | 0.507 | 231 | [39.1 – 41.1] | < 0.0001 |
|  | 1 month | 45.0 | 0.614 | 159 | [43.8 – 46.2] | < 0.0001 |
|  | 3 months | 45.5 | 0.729 | 108 | [44.1 – 47.0] | < 0.0001 |
| Suicidal Ideation (SIDAS) | Baseline | 8.70 | 0.852 | 233 | [7.02 – 10.38] | < 0.0001 |
|  | 1 month | 4.54 | 0.711 | 179 | [3.14 – 5.94] | < 0.0001 |
|  | 3 months | 4.06 | 0.859 | 114 | [2.36 – 5.76] | < 0.0001 |

**S9. Time Contrasts for Each Outcome**

| **Outcome** | **Contrast** | **Estimate** | **Standard Error** | **DF** | **Adjusted p-value** |
| --- | --- | --- | --- | --- | --- |
| Distress (YP-CORE) | B – 1M | 4.78 | 0.558 | 145.4 | < 0.0001 |
|  | B – 3M | 5.31 | 0.723 | 115.4 | < 0.0001 |
|  | 1M – 3M | 0.53 | 0.685 | 95.4 | 1.0000 |
| Depression (PHQ) | B – 1M | 3.43 | 0.396 | 148 | < 0.0001 |
|  | B – 3M | 3.17 | 0.461 | 116 | < 0.0001 |
|  | 1M – 3M | -0.27 | 0.476 | 109 | 1.0000 |
| Anxiety (GAD) | B – 1M | 3.00 | 0.429 | 149.8 | < 0.0001 |
|  | B – 3M | 3.07 | 0.496 | 113.1 | < 0.0001 |
|  | 1M – 3M | 0.06 | 0.471 | 93.8 | 1.0000 |
| Hopelessness (BHS) | B – 1M | 1.34 | 0.218 | 150 | < 0.0001 |
|  | B – 3M | 1.60 | 0.262 | 123 | < 0.0001 |
|  | 1M – 3M | 0.27 | 0.268 | 109 | 1.0000 |
| Stigma: Negative Stereotypes (AMIS) | B – 1M | -0.09 | 0.209 | 135.7 | 1.0000 |
|  | B – 3M | 0.29 | 0.293 | 128.9 | 0.9520 |
|  | 1M – 3M | 0.39 | 0.264 | 89.2 | 0.4339 |
| Stigma: Recovery and Outcomes (AMIS) | B – 1M | 0.09 | 0.217 | 147 | 1.0000 |
|  | B – 3M | 0.26 | 0.264 | 123 | 0.9991 |
|  | 1M – 3M | 0.17 | 0.240 | 92 | 1.0000 |
| Loneliness (ULS) | B – 1M | 0.53 | 0.124 | 152.5 | 0.0001 |
|  | B – 3M | 0.87 | 0.171 | 115.7 | < 0.0001 |
|  | 1M – 3M | 0.34 | 0.164 | 98.7 | 0.1295 |
| Social Support: Friends (MSPSS) | B – 1M | -0.52 | 0.395 | 138.4 | 0.5621 |
|  | B – 3M | -4.56 | 0.499 | 103.3 | 1.0000 |
|  | 1M – 3M | 0.07 | 0.513 | 95.1 | 1.0000 |
| Social Support: Family (MSPSS) | B – 1M | -0.33 | 0.351 | 149 | 1.0000 |
|  | B – 3M | -0.69 | 0.490 | 128 | 0.4922 |
|  | 1M – 3M | -0.35 | 0.472 | 110 | 1.0000 |
| Expectancies | B – 1M | -0.02 | 0.180 | 147.1 | 1.0000 |
|  | B – 3M | 0.38 | 0.237 | 120.3 | 0.3355 |
|  | 1M – 3M | 0.40 | 0.224 | 97.9 | 0.2397 |
| Global Physical Health (PROMIS) | B – 1M | -1.69 | 0.528 | 151 | 0.0050 |
|  | B – 3M | -2.56 | 0.646 | 108 | 0.0004 |
|  | 1M – 3M | -0.87 | 0.655 | 101 | 0.5581 |
| Global Mental Health (PROMIS) | B – 1M | -4.87 | 0.590 | 149 | < 0.0001 |
|  | B – 3M | -5.38 | 0.752 | 116 | < 0.0001 |
|  | 1M – 3M | -0.51 | 0.828 | 106 | 1.0000 |
| Suicidal Ideation (SIDAS) | B – 1M | 4.16 | 0.647 | 156.3 | < 0.0001 |
|  | B – 3M | 4.64 | 1.010 | 146.1 | < 0.0001 |
|  | 1M – 3M | 0.48 | 0.795 | 98.3 | 1.0000 |
|  |  |  |  |  |  |
